# Effects of a 12-Week Multifaceted Wearable-Based Program for People With Knee Osteoarthritis: Randomized Controlled Trial

**DOI:** 10.2196/19116

**Published:** 2020-07-03

**Authors:** Linda C Li, Lynne M Feehan, Hui Xie, Na Lu, Christopher D Shaw, Diane Gromala, Siyi Zhu, J Antonio Aviña-Zubieta, Alison M Hoens, Cheryl Koehn, Johnathan Tam, Stephanie Therrien, Anne F Townsend, Gregory Noonan, Catherine L Backman

**Affiliations:** 1 Department of Physical Therapy University of British Columbia Vancouver, BC Canada; 2 Arthritis Research Canada Richmond, BC Canada; 3 Faculty of Health Sciences Simon Fraser University Burnaby, BC Canada; 4 School of Interactive Art & Technology Simon Fraser University Burnaby, BC Canada; 5 Department of Rehabilitation Medicine West China Hospital Sichuan University Chengdu China; 6 Rehabilitation Medicine Key Laboratory of Sichuan Province West China Hospital Sichuan University Chengdu China; 7 Department of Medicine University of British Columbia Vancouver, BC Canada; 8 Arthritis Consumer Experts Vancouver, BC Canada; 9 Division of Health Research Faculty of Health & Medicine Lancaster University Lancashire United Kingdom; 10 Mary Pack Arthritis Program Vancouver General Hospital Vancouver, BC Canada; 11 Department of Occupational Science & Occupational Therapy University of British Columbia Vancouver, BC Canada

**Keywords:** physical activity, counseling, knee osteoarthritis, physiotherapy, wearables

## Abstract

**Background:**

Current guidelines emphasize an active lifestyle in the management of knee osteoarthritis (OA), but up to 90% of patients with OA are inactive. In a previous study, we demonstrated that an 8-week physiotherapist (PT)-led counseling intervention, with the use of a Fitbit, improved step count and quality of life in patients with knee OA, compared with a control.

**Objective:**

This study aimed to examine the effect of a 12-week, multifaceted wearable-based program on physical activity and patient outcomes in patients with knee OA.

**Methods:**

This was a randomized controlled trial with a delay-control design. The immediate group (IG) received group education, a Fitbit, access to FitViz (a Fitbit-compatible app), and 4 biweekly phone calls from a PT over 8 weeks. Participants then continued using Fitbit and FitViz independently up to week 12. The delay group (DG) received a monthly electronic newsletter in weeks 1 to 12 and started the same intervention in week 14. Participants were assessed in weeks 13, 26, and 39. The primary outcome was time spent in daily moderate-to-vigorous physical activity (MVPA; in bouts ≥10 min) measured with a SenseWear Mini. Secondary outcomes included daily steps, time spent in purposeful activity and sedentary behavior, Knee Injury and OA Outcome Score, Patient Health Questionnaire-9, Partners in Health Scale, Theory of Planned Behavior Questionnaire, and Self-Reported Habit Index.

**Results:**

We enrolled 51 participants (IG: n=26 and DG: n=25). Compared with the IG, the DG accumulated significantly more MVPA time at baseline. The adjusted mean difference in MVPA was 13.1 min per day (95% CI 1.6 to 24.5). A significant effect was also found in the adjusted mean difference in perceived sitting habit at work (0.7; 95% CI 0.2 to 1.2) and during leisure activities (0.7; 95% CI 0.2 to 1.2). No significant effect was found in the remaining secondary outcomes.

**Conclusions:**

A 12-week multifaceted program with the use of a wearable device, an app, and PT counseling improved physical activity in people with knee OA.

**Trial Registration:**

ClinicalTrials.gov NCT02585323; https://clinicaltrials.gov/ct2/show/NCT02585323

## Introduction

### Background

Arthritis is the most common cause of severe chronic pain and disability worldwide [1,2]. Analysis by the Arthritis Alliance of Canada estimates a new diagnosis of osteoarthritis (OA) every 60 seconds [3]. Current evidence supports the use of physical activity to manage OA because of its benefits on pain, mobility, and quality of life [4-6]. Guidelines by the OA Research Society International recommend the use of physical activity and therapeutic exercise as first-line treatment for knee OA [7]. Canadian physical activity guidelines recommend ≥150 min a week of moderate-to-vigorous physical activity (MVPA), performed in bouts of ≥10 min [8]. A study using accelerometers, however, found that over 90% of people with knee OA did not meet the physical activity guidelines [9]. This concurs with a systematic review that only 13% of people with OA accumulated ≥150 min per week of MVPA in bouts of ≥10 min [10].

Several modifiable factors are associated with low physical activity participation in patients with arthritis. These include lack of motivation [11], doubts about the effectiveness of exercise [12], and lack of health professional advice [13]. To promote an active lifestyle, the use of activity tracking devices has been explored based on the assumption that providing direct feedback on the amount of physical activity encourages people to meet specific targets. A systematic review of 14 randomized controlled trials (RCTs) of accelerometer-based interventions reported a small effect on physical activity participation (standardized mean difference 0.26; 95% CI 0.04 to 0.49). Notably, consumer-grade accelerometers promote physical activity behaviors through the use of behavioral change techniques [14], such as goal setting, self-monitoring, feedback, and rewards [15]. However, effective techniques such as action planning and problem-solving are absent from the use of these devices alone [15]. These techniques require contact with a health professional with counseling experience.

Our previous study [16] and Lyons et al [17] have demonstrated the feasibility of a physical activity counseling program with the use of a Fitbit (Fitbit Inc), a consumer-grade wearable device. Our subsequent study showed that an 8-week physiotherapist (PT)-led counseling program improved step count and quality of life in people with knee OA, compared with a control [18].

### Study Aim

In this study, we aimed to assess the effect of a 12-week multifaceted intervention on improving activity participation in people with knee OA. Our primary hypothesis was that, compared with controls, those who received the program would increase mean daily MVPA time as determined by an objective measure. In addition, we explored the effect of the program on OA disease status, depressive symptoms, perceived habitual behaviors, and psychological constructs of being active.

## Methods

### Study Design

**Figure 1:**
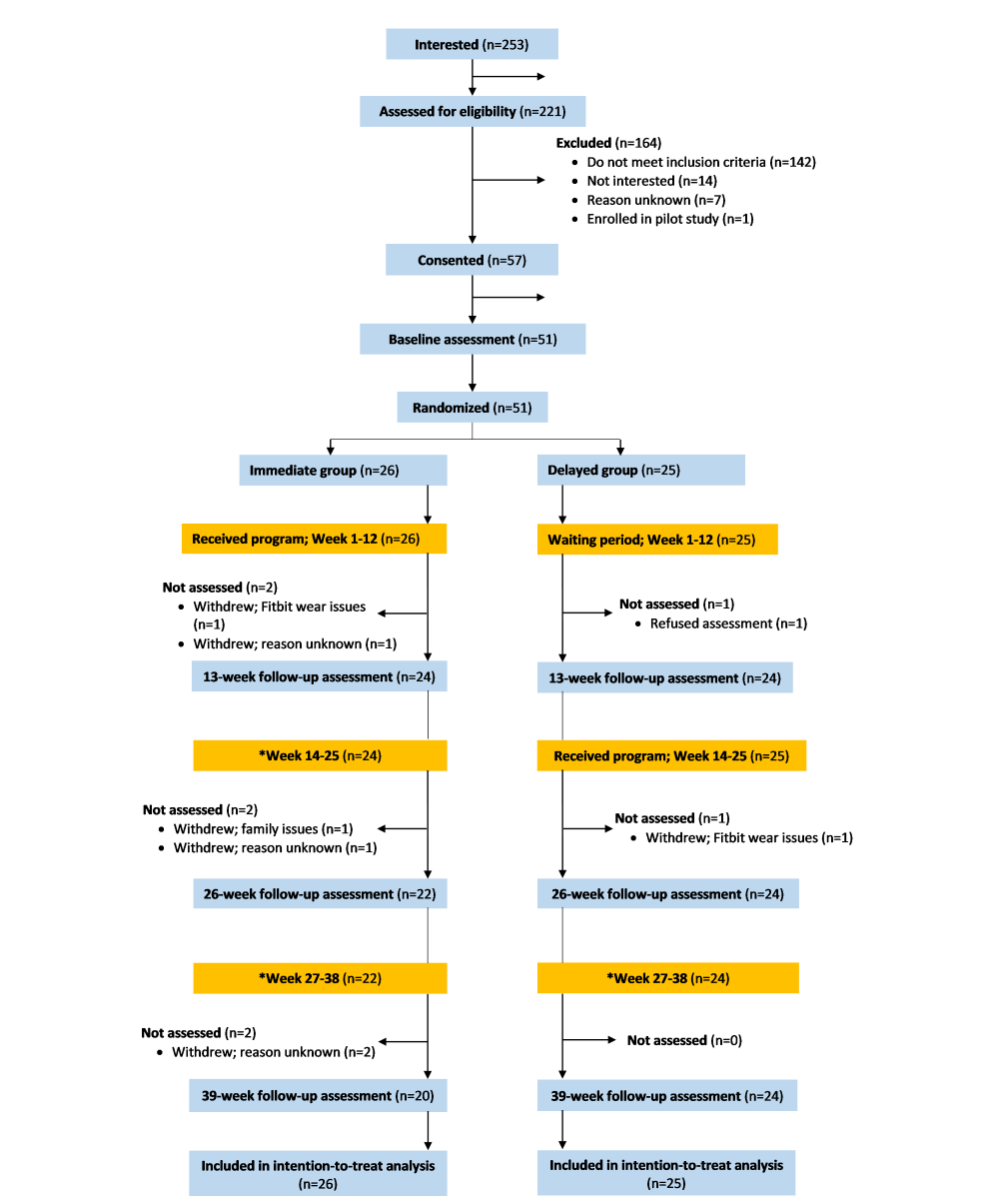
Consolidated Standards of Reporting Trials flowchart.

The Supporting Physical activity & Reducing sedentary behaviour in Arthritis (SuPRA) project was a proof-of-concept RCT with a delay-control design. Participants were randomly assigned to start the intervention either immediately (*immediate group* [IG]) or 14 weeks (*delay group* [DG]) after completing the baseline assessment. All participants were reassessed in weeks 13 (primary end point), 26, and 39 (Figure 1).

### Participants

Participants were recruited from the Mary Pack Arthritis Program (Vancouver Coastal Health Authority) and the Fraser Health Authority in British Columbia, Canada. We also posted study information on Facebook, Twitter, Kajiji, and Craigslist. Individuals were eligible if they had a diagnosis of knee OA or met 2 criteria for early OA [19]. People with other chronic musculoskeletal conditions or contraindications to be physically active without medical supervision were excluded (Textboxes 1 and 2).

**Inclusion criteria.**

**Inclusion criteria**

- Patients who had a physician-confirmed diagnosis of knee osteoarthritis or were aged ≥50 years and had felt pain or discomfort in or around the knee during the previous year lasting >28 separate or consecutive days [19]
- Patients who had no previous diagnosis of rheumatoid arthritis, psoriatic arthritis, ankylosing spondylitis, polymyalgia rheumatica, connective tissue diseases, fibromyalgia, or gout
- Patients who had no history of using disease-modifying antirheumatic drugs or gout medications
- Patients who had no prior knee arthroplasty and not on a waiting list for total knee or hip replacement surgery
- Patients who did not have surgery in the back, hip, knee, foot, or ankle joints in the past 12 months
- Patients who had no history of acute injury to the knee in the past 6 months
- Patients who had an email address and access to the internet daily
- Patients who were able to attend a 1.5-hour group education session

**Exclusion criteria.**

**Exclusion criteria**

- Patients who had previously used a physical activity wearable tracker
- Patients who received a steroid injection in a knee in the last 6 months
- Patients who received a hyaluronate injection in a knee in the last 6 months
- Patients who used medication that may impair activity tolerance (eg, beta blockers)
- Patients who faced a level of risk by exercising as identified by the Physical Activity Readiness Questionnaire [20]. If a participant did not pass the Physical Activity Readiness Questionnaire, a physician’s note was requested to determine the eligibility

### Randomization and Blinding

After completing the baseline measures, participants were randomly assigned to the IG or the DG in a 1:1 allocation ratio. Randomization was performed by a research staff not involved in the study using numbers generated by SAS version 9.4 (SAS Institute Inc) in variable block sizes to ensure allocation concealment. Participants were not blinded as they knew whether they received the program during the intervention period. Daily MVPA time (the primary outcome) was measured with a wearable multisensor device couriered to the participant to wear for 7 days. All research personnel processing the objectively measured physical activity data were blinded to the participants’ group assignment. Self-reported measures were completed by the participant via a web-based questionnaire.

### Intervention

The intervention has 3 components: (1) an in-person session with 20 min of group education and 30 min of individual counseling with a PT, (2) the use of a Fitbit Flex-2 wristband, and (3) PT counseling by phone to review physical activity goals (20-30 min). The in-person session was held in a meeting room at either the Mary Park Arthritis Centre, Fraser Health Authority, or Arthritis Research Canada. Fitbit is a consumer-grade wearable device that tracks and displays steps walked, gross level of physical exertion, and time spent being active. It is easy for users to put on and remove for charging. Fitbit Flex-2 is splash proof but cannot be used during water-based activities (eg, shower, swimming). Participants may record activities not captured by the device on the Fitbit website. Participants could view their physical activity goal attainment on FitViz, a new Fitbit-compatible web-based app developed for this study [21].

All 8 study PTs completed a 2-day basic training in motivational interviewing at the University of British Columbia [22]. In addition, they attended an orientation session, received a counseling guide, and shadowed at least one education and counseling session before they were paired with a participant.

Participants in the IG were scheduled to attend a PT-led group education session that focused on physical activity in OA management and strategies to manage joint symptoms. Next, each participant was paired with the next available study PT. During the individual counseling, PTs used the Brief Action Planning approach [23] to guide participants to set *specific, measurable, attainable, relevant, and time-bound* (SMART) physical activity goals. An example of a SMART goal is “attending a pool exercise class in a community center every Tuesday and Saturday for the next three months.”

Study PTs then set the parameters on their assigned participants’ FitViz accounts based on the participant’s goals. These parameters included the following: (1) the upper and lower limits of intensity and duration of MVPA (ie, to promote physical activity based on the participant’s goal), (2) the duration when a sedentary behavior should be interrupted (ie, to promote *less sitting*), and (3) the rest time in between sessions of MVPA (ie, to promote pacing).

During weeks 1 to 8, the study PT remotely reviewed participants’ progress on FitViz and counseled them to modify their physical activity goals via 4 biweekly phone calls. A counseling guide was provided, and the discussion was documented by the PT. During weeks 9 to 12, participants continued using their Fitbit and FitViz but had no counseling calls with their PTs. However, participants could email their PTs if there were questions related to being physically active. At the end of the program, participants could keep their Fitbit and FitViz account.

The DG received the same intervention in week 14. During the waiting period, they received monthly emails of arthritis news that were unrelated to physical activity.

### Outcome Measures

The primary outcome measure was mean daily MVPA time measured with SenseWear Mini (BodyMedia Inc). SenseWear integrates triaxial accelerometer data, physiological sensor data, and personal demographic information to provide estimates of steps and energy expenditure. A strong relationship has been found between SenseWear and indirect calorimetry measures of energy expenditure for activities of daily living (Pearson *r*=0.85) [24]. The device can be worn 24 hours a day and can capture a full picture of physical activity and off-body time throughout the day [25]. An important feature of SenseWear is its ability to differentiate between sedentary and light physical activities [26]; hence, it is an ideal instrument to assess both active and sedentary behaviors.

Participants wore a SenseWear Mini over the triceps for 7 days at each assessment. Almeida et al [27] determined that a minimum of 4 days of wear was required to reliably assess energy expenditure from different levels of physical activity in people with rheumatoid arthritis (intraclass correlation coefficient >0.80). We calculated the average MVPA accumulated in bouts (min per day). A bout was defined as ≥10 min at the level of ≥3 metabolic equivalent of the task (MET), with an allowance for interruption of up to 2 min below the threshold [28].

The secondary outcomes included the following:

1. Average daily time in purposeful activity performed in ≥4 MET in bouts of ≥10 min, with allowance for interruption of up to 2 min below the threshold (eg, brisk walking) [29].
2. Average daily step count.
3. Average daily time in sedentary behavior performed in ≤1.5 MET in bouts of ≥20 min during waking hours [30-33].
4. Knee Injury and OA Outcome Score (KOOS) [34,35].
5. Partners in Health Scale (PIHS) [36].
6. Theory of Planned Behavior Questionnaire [37,38].
7. Patient Health Questionnaire-9 (PHQ-9) [39].
8. Self-Reported Habit Index [40,41].

We used SenseWear to measure purposeful activity time, steps, and sedentary behavior time. The KOOS consists of 5 subscales: *pain*, *symptoms*, *activities of daily living*, *sports/recreation*, and *knee-related quality of life*. It was originally developed for people recovering from injuries such as the anterior cruciate ligament and meniscus injury and was validated in patients with OA [34,35]. The PIHS is a 12-item measure designed to assess self-efficacy, knowledge of health conditions and treatment, and self-management behavior, such as adopting a healthy lifestyle (Cronbach α=.82) [36]. Motivation for engaging in physical activity was measured using the Rhodes 7-point Likert-type Theory of Planned Behavior questionnaire [37,38]. The questionnaire consists of 16 items measuring all components of the theory. Previous studies using this measure showed good predictive validity and internal consistency in adult populations [37,38].

The PHQ-9 consists of 9 questions that correspond to the diagnostic criteria for major depressive disorder. A score greater than 11 indicates a major depressive disorder [39]. The Self-Reported Habit Index is a 12-item scale, rated on a 7-point Likert scale, which measures characteristics of habitual behavior (reliability minimum α=.81) [40,41]. We asked participants to rate their strength of habit for 3 specific activity-related behaviors: sitting during leisure time at home, sitting during usual occupational activities, and walking outside for 10 min. A higher score indicates a stronger habit or behavior that is done frequently and automatically. Demographic variables and comorbid conditions were collected at baseline.

### Adverse Event and Intervention Fidelity Monitoring

We tracked adverse events (falls as well as cardiovascular and musculoskeletal events) related to their physical activity [42] in the follow-up questionnaire at weeks 13, 26, and 39. Participants were deemed adhering to the 12-week intervention protocol if they (1) attended the education session, (2) used their Fitbit ≥5 days per week in ≥11 weeks, and (3) participated in ≥3 of 4 counseling calls. We monitored participants’ Fitbit wear using FitViz, which wirelessly synchronized physical activity data recorded by a Fitbit 150 times per hour [43]. We calculated the percentage of participants meeting each criterion and all 3 criteria.

### Data Analysis

Descriptive analysis was used to summarize participant characteristics and comorbid conditions. We performed an intention-to-treat analysis using SAS version 9.4. The analysis of covariance (ANCOVA) was used for the main analysis to estimate an adjusted mean difference comparing time in MVPA (primary outcome) at 13 weeks between groups, adjusting for baseline MVPA and blocking. In a secondary analysis, we used a longitudinal mixed effects model that allowed us to additionally examine the intervention effects at 26 and 39 weeks after intervention initiation. The mixed effects models included the following variables as fixed effects: (1) the randomization group indicator for baseline difference, (2) a set of indicator variables for follow-up assessment time points (weeks 13, 26, and 39) to account for secular trend, and (3) a set of indicator variables for the lengths of time since intervention initiation (weeks 12, 25, or 38) to estimate intervention effects after these amounts of time postintervention initiation. The models additionally included participants as random effects to account for the repeated measures nature of the data. We used the sandwich estimators for linear mixed models [44] to compute empirical standard errors that were robust to model specifications. We examined the secondary outcomes at 13 weeks using ANCOVA and over weeks 26 and 39 using the mixed effects models mentioned earlier.

### Sample Size

We estimated that our collaboration with health authorities and patient groups allowed the study to recruit 60 eligible participants within 12 months. Our previous study of a similar physical activity counseling program resulted in an estimated MVPA time of 75.5 min per day (SD 54.3) in the intervention group and 50.0 min per day (SD 46.8) in the controls [18]. Assuming approximately 15% attrition, we anticipated that 50 of the 60 participants would complete the study. With a sample size of 50, we would have 74% power at an α level of 0.1 (via a one-sided test).

### Ethics

The research protocol was approved by the University of British Columbia Behavioural Research Ethics Board (application number: H15-02038) and published in ClinicalTials.gov (NCT02585323).

## Results

### Baseline Characteristics

In the years 2017 to 2019, 253 people indicated an interest to participate, and 221 met the eligibility criteria (Figure 1). Of these, we recruited 51 participants (IG: 23/26, 88% were women; DG: 19/25, 76% were women). Both groups were similar in age (IG: mean 65.0, SD 8.3 years; DG: mean 64.8, SD 9.0 years) and BMI (IG: mean 29.8, SD 9.0 kg/m^2^; DG: mean 28.9, SD 6.2 kg/m^2^). Approximately 55% (28/51) of the participants did not meet the Canadian physical activity guidelines at baseline (Table 1).

**Table 1:** Baseline characteristics of participants.

Variables		All (N=51)	Immediate group (n=26)	Delay group (n=25)
Women, n (%)		42 (82)	23 (89)	19 (76)
Age (years), mean (SD)		64.9 (8.5)	65.0 (8)	64.8 (9)
**Marital status, n (%)**				
	Married/common law	30 (59)	18 (69)	12 (48)
	Separated/divorced	10 (20)	5 (19)	5 (20)
	Widowed/never married/other	11 (22)	3 (12)	8 (32)
University degree or trades certificate, n (%)		25 (49)	14 (54)	11 (44)
**Gross annual household income (US $), n (%)**				
	≤24,000	2 (4)	0 (0)	2 (8)
	24,001-40,000	6 (12)	2 (8)	4 (16)
	40,001-60,000	9 (18)	4 (15)	5 (20)
	60,001-80,000	8 (16)	4 (15)	4 (16)
	80,001-100,000	7 (14)	5 (19)	2 (8)
	>100,000	7 (14)	3 (12)	4 (16)
	No answer	12 (24)	8 (31)	4 (16)
**Diagnosed with OA^a^, n (%)**				
	Yes	37 (73)	20 (77)	17 (68)
	No, but met the likely OA criteria	14 (28)	6 (23)	8 (32)
**In general, would you say your health is,** **n (%)**				
	Excellent	6 (12)	1 (4)	5 (20)
	Very good	14 (27)	10 (39)	4 (16)
	Good	22 (43)	11 (42)	11 (44)
	Fair	8 (16)	3 (12)	5 (20)
	Poor	1 (2)	1 (4)	0 (0)
**Compared with 1 year ago, how would you rate your health in general now? n (%)**				
	Much better	3 (6)	1 (4)	2 (8)
	Somewhat better	8 (16)	4 (15)	4 (16)
	About the same	24 (47)	11 (42)	13 (52)
	Somewhat worse	16 (31)	10 (39)	6 (24)
	Much worse	0 (0)	0 (0)	0 (0)
Number of comorbid conditions, median (25th, 75th percentile)		3.0 (2.0, 5.0)	4.0 (3.0, 5.0)	3.0 (2.0, 4.0)
BMI (kg/m^2^), mean (SD)		29.4 (7.7)	29.8 (9.0)	28.9 (6.2)
Participants did not meet the Canadian physical activity guideline (≥150 min of MVPA^b^ in bouts of ≥10 min per week), n (%)		28 (55)	15 (58)	13 (52)

^a^OA: osteoarthritis.

^b^MVPA: moderate-to-vigorous physical activity.

### Comparison of the Immediate Group With Delay Group

**Figure 2:**
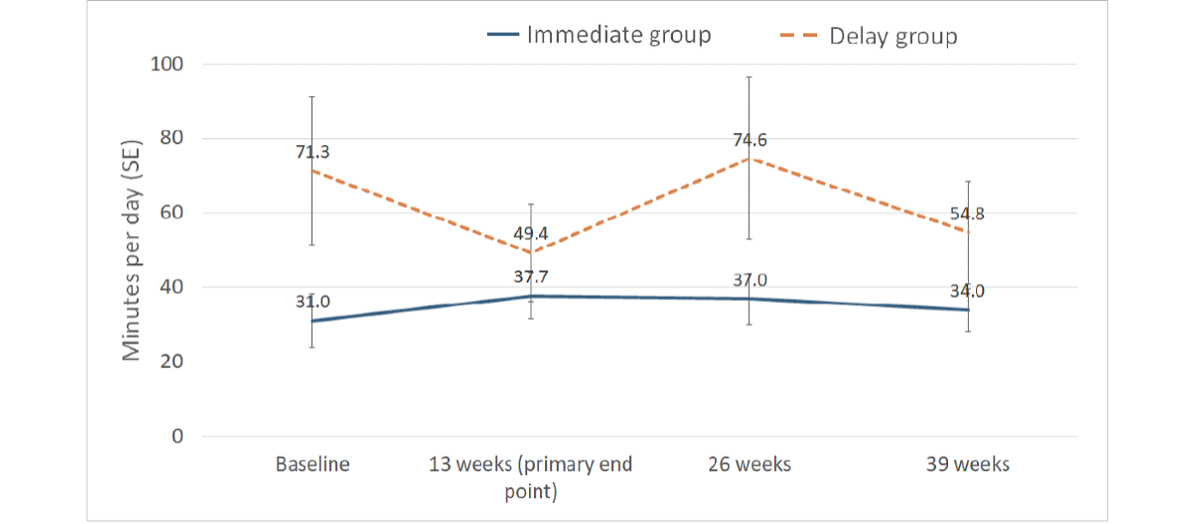
Time in moderate-to-vigorous physical activity.

**Figure 3:**
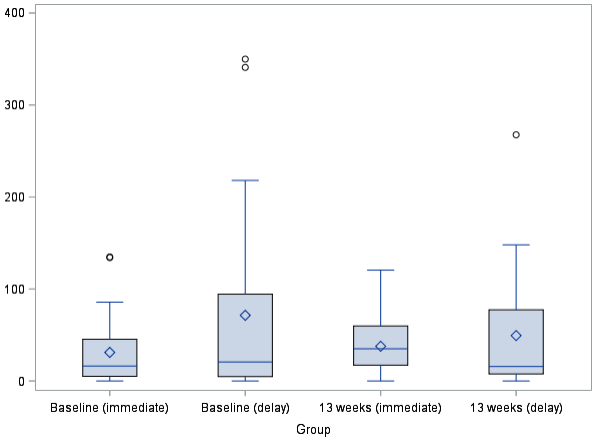
Boxplot of time in moderate-to-vigorous physical activity at baseline and 13 weeks.

Table 2 and Figure 2 present the results of the primary outcome from 4 time points. At baseline, the mean MVPA time was 31.0 min per day (SD 37.3) for the IG and 71.3 min per day (SD 99.8) for the DG. The DG accumulated significantly more MVPA time—2 outliners accumulated a mean of >300 min per day (Figure 3). At 13 weeks (the primary end point), the IG accumulated a mean MVPA of 37.7 min per day (SD 30.5), whereas the DG had 49.4 min per day (SD 63.6). The adjusted mean difference in time spent in MVPA between groups following the intervention at 13 weeks was 13.1 min per day (95% CI 1.6 to 24.5), favoring the IG. Analyses adjusted for blocking yielded nearly identical results; thus, it was removed from subsequent analyses. A secondary analysis using a mixed effects model revealed smaller intervention effects at 12 weeks (9.4 min per day; 95% CI −3.0 to 21.7), 25 weeks (−3.0 min per day; 95% CI −34.9 to 29.0), and 38 weeks (0.2 min per day; 95% CI −44.0 to 44.4) postprogram initiation (Table 3).

**Table 2:** Participant outcomes and results of the primary analysis.

Measures		Immediate group, mean (SD)				Delay group, mean (SD)				Adjusted difference in mean change at T0-T1	
		Baseline (T0; n=26)	13 weeks (T1; n=24)	26 weeks (T2; n=22)	39 weeks (T3; n=20)	Baseline (T0; n=25)	13 weeks (T1; n=24)	26 weeks (T2; n=22)	39 weeks (T3; n=23)	Mean difference (95% CI)	*P* value
Time in MVPA^a,b^ (min)		31.0 (37.3)	37.7 (30.5)	37.0 (32.3)	34.0 (25.2)	71.3 (99.8)	49.4 (63.6)	74.6 (102.1)	54.8 (66.2)	13.1 (1.6 to 24.5)	.03
Time in purposeful activity^c^ (min)		11.1 (19.5)	13.3 (20.0)	13.7 (18.8)	12.8 (16.8)	42.1 (80.2)	23.1 (37.1)	36.5 (62.6)	22.0 (42.3)	1.6 (−3.0 to 6.1)	.50
Daily steps		6294.0 (3418.0)	7133.3 (3603.3)	6381.6 (3492.0)	5845.3 (2575.5)	7030.1 (3921.6)	6232.7 (3086.1)	8162.3 (6642.3)	7445.1 (4713.2)	1106.5 (−19.9 to 2232.9)	.05
Sedentary time^d^ (min)		567.5 (183.1)	531.4 (173.5)	492.5 (156.6)	502.8 (135.3)	551.1 (234.9)	558.3 (224.9)	499.5 (248.9)	483.1 (225.4)	−29.5 (−75.8 to 16.7)	.21
**Knee Injury and Osteoarthritis Outcome Score (0-100; higher=better)**											
	Symptoms	68.5 (10.7)	69.3 (12.7)	65.4 (45.8)	68.4 (16.3)^e^	65.7 (12.3)	66.9 (14.9)^f^	72.2 (16.8)^g^	72.5 (13.3)^g^	−1.4 (−7.8 to 4.9)	.66
	Pain	72.6 (13.5)	73.1 (15.3)	72.5 (18.3)	72.1 (19.8)^e^	65.1 (13.7)	65.9 (15.6)^f^	74.8 (15.4)^g^	72.8 (13.2)^g^	2.5 (−4.2 to 9.5)	.49
	Activity of daily living	75.5 (14.7)	75.0 (13.1)	77.7 (18.9)	74.8 (20.7)^e^	72.2 (15.8)	70.3 (16.9)^f^	80.3 (13.1)^g^	80.0 (14.0)^g^	2.8 (−3.3 to 8.8)	.37
	Sports and recreation	47.9 (23.7)	47.1 (22.4)	50.5 (30.1)	51.6 (30.4)^e^	46.8 (25.4)	52.5 (22.7)^f^	62.5 (22.5)^g^	55.8 (26.3)^g^	−3.8 (−14.9 to 7.2)	.50
	Quality of life	44.0 (16.0)	48.7 (17.5)	49.4 (15.7)	49.3 (19.1)^e^	47.5 (16.0)	46.9 (13.6)^f^	54.7 (14.7)^g^	54.4 (14.6)^g^	1.4 (−5.0 to 7.9)	.66
Partners in Health (0-96; higher=better)		76.8 (11.0)	76.7 (11.5)	78.9 (9.0)	81.0 (7.0)^e^	78.0 (12.0)	81.6 (9.6)^f^	82.6 (9.3)^g^	82.6 (10.0)^g^	−2.3 (−6.6 to 1.9)	.28
Patient Health Questionnaire-9 (0-27; lower=better)		5.2 (4.6)	4.0 (3.0)	3.6 (3.3)	4.2 (4.1)^e^	5.4 (5.3)	4.7 (4.9)^f^	4.5 (5.2)^g^	4.5 (5.3)^g^	−0.4 (−1.7 to 0.8)	.47
**Self-Reported Habit Index (1-7; higher=stronger habit)**											
	Sitting at work subscale	5.0 (1.4)	5.2 (1.6)	4.7 (2.1)	5.0 (1.9)^e^	4.5 (2.1)	4.4 (2.0)^f^	4.3 (2.1)^g^	4.1 (2.1)^g^	0.7 (0.2 to 1.2)	.004
	Sitting at leisure subscale	4.8 (1.1)	5.1 (1.1)	5.0 (1.1)	5.5 (1.1)^e^	5.1 (1.4)	4.7 (1.6)^f^	4.4 (1.9)^g^	4.7 (1.7)^g^	0.7 (0.2 to 1.2)	.006
	Walking subscale	4.3 (1.8)	4.4 (1.6)	4.6 (1.8)	4.7 (1.8)^e^	4.8 (2.0)	4.6 (2.0)^f^	4.8 (1.6)^g^	4.6 (1.8)^g^	0.3 (−0.3 to 0.9)	.27
**Theory of Planned Behavior Questionnaire (1-7; higher=more positive)**											
	Attitude toward physical activity	6.0 (0.6)	5.9 (0.7)	6.0 (0.5)	6.0 (0.5)^e^	6.1 (0.6)	6.1 (0.7)^f^	6.2 (0.6)^g^	6.1 (0.7)^g^	−0.1 (−0.4 to 0.2)	.63
	Subjective norm	6.2 (0.6)	6.2 (0.8)	6.1 (0.8)	6.2 (1.1)^e^	6.3 (0.8)	6.2 (0.7)^f^	6.3 (0.8)^g^	6.3 (0.7)^g^	0.2 (−0.1 to 0.5)	.13
	Perceived control	5.8 (1.0)	5.6 (1.4)	5.6(1.5)	5.7 (1.4)^e^	6.1 (0.7)	5.8 (1.2)^f^	6.2 (1.0)^g^	6.2 (0.9)^g^	0.1 (−0.6 to 0.8)	.80
	Intention	6.2 (0.8)	5.9 (1.0)	5.7 (1.1)	5.8 (0.8)^e^	6.3 (0.8)	6.4 (0.6)^f^	6.4 (0.6)^g^	6.3 (0.9)^g^	0.1 (−0.1 to 0.3)	.22

^a^MVPA: moderate-to-vigorous physical activity.

^b^MVPA was performed at ≥3 metabolic equivalent of tasks and in bouts ≥10 min.

^c^Purposeful activity was performed at ≥4 METs and in bouts ≥10 min.

^d^Sedentary behavior was performed at ≤1.5 METs in bouts ≥20 min.

^e^n=19.

^f^n=22.

^g^n=24.

**Table 3:** Results of mixed effects models.

Measures		Intervention effect postprogram initiation (95% CI)			Secular trend (95% CI)		
		12 weeks	25 weeks	38 weeks		T2^a^ effect vs T1^b^	T3^c^ effect vs T1
Time in MVPA^d,e^ (min)		9.4 (−3.0 to 21.7)	−3.0 (−34.9 to 29.0)	0.2 (−44.0 to 44.4)		11.9 (−12.4 to 36.1)	6.4 (−29.7 to 42.5)
Time in purposeful activity^f^ (min)		1.5 (−8.4 to 11.5)	−9.0 (−33.5 to 15.5)	−6.8 (−42.3 to 28.6)		10.0 (−8.0 to 28.0)	6.9 (−21.3 to 35.1)
Daily steps		1461.2 (433.8 to 2488.6)	715.0 (−1995.9 to 3425.9)	71.4 (−4063.6 to 4206.3)		101.7 (−2142.6 to 2346.0)	336.9 (−3261.7 to 3935.4)
**Sedentary time^g^ (min)**		−32.2 (−78.1 to 13.7)	−62.6 (−162.8 to 37.6)	−63.4 (−217.6 to 90.8)		−19.8 (−83.7 to 44.1)	−7.6 (−117.0 to 101.9)
	Symptoms	−2.2 (−8.2 to 3.4)	−12.4 (−22.6 to −2.2)	−21.1 (−36.8 to −5.4)		6.1 (0.2 to 11.9)	16.5 (6.2 to 26.9)
	Pain	0.3 (−6.2 to 6.8)	−7.8 (−19.7 to 4.1)	−15.1 (−33.1 to 3.0)		7.5 (1.3 to 13.8)	13.7 (2.0 to 25.4)
	Activity of daily living	1.1 (−5.0 to 7.1)	−4.6 (−16.2 to 7.0)	−13.2 (−32.0 to 5.6)		8.1 (2.7 to 13.6)	13.5 (2.1 to 24.9)
	Sports and recreation	−7.4 (−18.7 to 3.9)	−21.2 (−42.4 to 0.1)	−27.2 (−59.2 to 4.9)		16.5 (5.1 to 27.9)	23.6 (2.5 to 44.7)
	Quality of life	2.4 (−4.6 to 9.3)	−2.1 (−13.6 to 9.4)	−6.9 (−24.8 to 11.0)		4.8 (−3.2 to 12.7)	9.0 (−3.2 to 21.2)
Partners in Health (0-96; higher=better)		−1.7 (−5.7 to 2.4)	−3.8 (−10.9 to 3.3)	−4.2 (−15.2 to 6.8)		3.4 (−1.1 to 7.9)	5.6 (−2.7 to 13.9)
**Patient Health Questionnaire-9 (0-27; lower=better)**		−0.9 (−2.4 to 0.6)	−1.3 (−4.0 to 1.3)	−1.3 (−5.5 to 2.9)		0.1 (−1.2 to 1.5)	0.6 (−2.2 to 3.3)
	Sitting at work subscale	0.4 (−0.3 to 1.1)	0.3 (−1.1 to 1.6)	0.6 (−1.5 to 2.7)		−0.5 (−1.2 to 0.3)	−0.5 (−1.9 to 0.9)
	Sitting at leisure subscale	0.6 (0.1 to 1.0)	1.4 (0.5 to 2.4)	2.4 (0.9 to 3.9)		−1.0 (−1.6 to −0.4)	−1.5 (−2.5 to −0.4)
	Walking subscale	0.3 (−0.3 to 0.9)	0.4 (−0.6 to 1.4)	0.7 (−1.1 to 2.4)		0 (−0.6 to 0.5)	−0.3 (−1.4 to 0.8)
	Beliefs toward physical activity	−0.1 (−0.4 to 0.2)	−0.2 (−0.7 to 0.3)	−0.3 (−1.1 to 0.4)		0.2 (−0.1 to 0.4)	0.2 (−0.4 to 0.7)
	Subjective norm	0.1 (−0.3 to 0.5)	0.0 (−0.6 to 0.6)	0.1 (−0.9 to 1.1)		0.0 (−0.4 to 0.4)	0.0 (−0.6 to 0.6)
	Perceived control	0.0 (−0.6 to 0.7)	−0.4 (−1.6 to 0.7)	−0.7 (−2.5 to 1.1)		0.3 (−0.3 to 1.0)	0.8 (−0.3 to 2.0)
	Intention	−0.4 (−0.7 to −0.1)	−1.1 (−1.7 to −0.4)	−1.5 (−2.6 to −0.5)		0.4 (0.1 to 0.7)	1.0 (0.3 to 1.7)

^a^T2: 26-week assessment.

^b^T1: 13-week assessment.

^c^T3: 39-week assessment.

^d^MVPA: moderate-to-vigorous physical activity.

^e^MVPA was performed at ≥3 metabolic equivalent of tasks and in bouts of ≥10 min.

^f^Purposeful activity was performed at ≥4 METS and in bouts of ≥10 min.

^g^Sedentary behavior was performed at ≤1.5 METS in bouts of ≥20 min.

For the secondary outcome, a trend favoring the IG, but not statistically significant, was found from baseline to 13 weeks in purposeful activity time (1.6 min per day; 95% CI −3.0 to 6.1), step count (1106.5; 95% CI −19.9 to 2232.9), and sedentary time (−29.5 min per day; 95% CI −75.8 to 16.7). The results from the KOOS, PIHS, and PHQ-9 were also not statistically significant (Table 2). We found a small effect in perceived sitting habit while at work (0.7; 95% CI 0.2 to 1.2) or during leisure activities (0.7; 95% CI 0.2 to 1.2).

Secondary analysis demonstrated an effect attributable to being in the program for daily steps at 12 weeks (1461.2; 95% CI 433.8 to 2488.6; Table 3). Knee symptoms (measured by the KOOS symptoms subscale), perceived sitting habit during leisure activities, and intention to be physically active also showed statistically significant effects attributable to being in the program for different durations, although statistically significant secular trends were also observed over the assessment period.

### Intervention Adherence and Adverse Events

Intervention adherence in the IG was 100% (26/26) for education session attendance, 96% (25/26) for PT counseling phone calls, and 81% (21/26) for Fitbit use (Table 4). In all, 81% (21/26) of participants met all 3 fidelity criteria. Adherence rates were similar in the DG when participants received the program in week 13. During the 4 weeks when the PT counseling ended, 2 participants each from the IG and the DG contacted their study PT via email with further questions regarding their physical activity.

After starting the program, 10 of the 51 participants reported adverse events because of physical activity; of those, 7 reported muscle pain (IG: n=5 and DG: n=2). Falls were reported by 3 in the IG; of those, 2 fell while being physically active (1 had an ankle sprain). Three participants from the DG also reported a fall; 1 occurred while being physically active. Of the remaining 2 participants who had a fall, 1 sustained a vertebral compression fracture.

**Table 4:** Summary of intervention adherence.

Adherence criterion	Immediate group (n=26), n (%)	Delay group (n=25), n (%)	All (N=51), n (%)
Attended the initial session with group education and met with a physiotherapist to set physical activity goals	26 (100)	25 (100)	51 (100)
Completed ≥3 of 4 counseling phone calls with a physiotherapist	25 (96)	22 (88)	47 (92)
Met Fitbit use criteria^a^ ≥11 weeks out of the 12-week intervention period	21 (81)	20 (80)	41 (80)
Met 2 of 3 criteria	25 (96)	24 (96)	49 (96)
Met all 3 criteria	21 (81)	18 (72)	39 (77)

^a^Participants had steps recorded in their Fitbit ≥5 days per week.

## Discussion

### Principal Findings

More than 1 in 6 people in the United States are using wearable devices to monitor their health [45], but the integration of these tools in chronic disease management is at an early stage. This study demonstrated the potential of a multifaceted wearable-based program for promoting MVPA in people with knee OA. We found an effect in the adjusted mean difference in time spent in MVPA between groups after the 12-week program. Furthermore, the mixed effects model analysis suggests a significant effect in daily steps attributable to the program.

These results, however, should be viewed in the context that the DG accumulated significantly more daily MVPA time than the IG at baseline, and the observed effect was primarily driven by a decline in daily MVPA time in the DG at week 13. More than 80% of participants rated their health as “good,” “very good,” or “excellent” at baseline, suggesting that they might have few health constraints to be physically active. Nonetheless, the results extend those of our previous RCT on a similar program, whereby a significant improvement in participation in physical activity was found in people with knee OA at the end of an 8-week intervention [18]. We also found a small effect on the awareness of sitting habits at work and during leisure activities at 13 weeks. The reason for this observation is unclear, but it is likely too small to be clinically important.

Our results contribute to the literature on physical activity promotion in arthritis management. Recommendations by the European League Against Rheumatism endorse the use of behavior change techniques to promote physical activity among people with arthritis [46]. The 2019 American College of Rheumatology/Arthritis Foundation guidelines further highlight the involvement of health professionals, including PTs, to deliver nonpharmacological treatment [47]. The optimal approach for supporting an active lifestyle in people with arthritis remains to be unclear, but research in healthy adults has shown that low-dose health coaching had little effect on physical activity behavior [48]. Although PTs are skilled in exercise prescription, a recent survey in Canada revealed up to 71% of the respondents wished to acquire further training in physical activity counseling.[49] This suggests an opportunity for professional development for PTs to master skills in counseling techniques that match their patients’ readiness to acquire a health-related behavior. In our study, training on motivational interviewing and the opportunity to shadow a PT with experience in counseling participants are essential to the program. The PTs’ written record for each interaction with participants allowed us to ensure that counseling followed the Brief Action Planning approach. Participants continuing to use their Fitbits suggests that the behavior of self-monitoring was sustained even after the PT counseling ended. Future research can refine and compare different implementation strategies of physical activity counseling for this population.

### Strengths and Limitations

A strength of this study was intervention fidelity, with an overall intervention adherence of 81%. There is no consensus on what constitutes good intervention adherence, but 80% to 100% has been deemed as high fidelity in delivery [50]. Furthermore, more than 80% of participants adhered to Fitbit use over the 12-week period, indicating that it is feasible to transition individuals from a multifaceted program to a wearable-only intervention after the initial 8-week counseling from a PT.

This study has some limitations. With the use of a delay-control design in which the participants in the control arm received the program after a 13-week delay, the efficacy of the counseling program could only be unequivocally assessed at 13 weeks. At 26 and 39 weeks, both groups had already received the intervention, and there was an absence of a control group at these 2 time points, which can cause intervention effect estimates at 26 and 39 weeks to be less robust and more susceptible to small sample bias. Hence, the long-term effects of the program remain unclear. Furthermore, the results may not be generalizable to men because 82% of the participants were women.

### Conclusions

Supporting a physically active lifestyle is a core component of physiotherapy practice. The Exercise is Medicine initiative advocates for the creation and worldwide implementation of effective physical activity promotion strategies in treatment plans for patients [51]. With the ubiquitous use of wearables, health professionals can leverage the use of these tools to motivate, monitor, and counsel people with arthritis to reach physical activity goals. To this end, we have shown that a 12-week multifaceted counseling program, with the use of a wearable device, can improve physical activity participation in people with knee OA.

## Appendix

Multimedia Appendix 1 CONSORT checklist.

## Abbreviations

ANCOVA: analysis of covariance
CIHR: Canadian Institutes of Health Research
DG: delay group
IG: immediate group
KOOS: Knee Injury and Osteoarthritis Outcome Score
MET: metabolic equivalent of task
MVPA: moderate-to-vigorous physical activity
OA: osteoarthritis
PHQ-9: Patient Health Questionnaire-9
PIHS: Partners in Health Scale
PT: physiotherapist
RCT: randomized controlled trial
SMART goal: specific, measurable, attainable, relevant, and time-bound goal
SuPRA: Supporting Physical activity & Reducing sedentary behaviour in Arthritis

## References

[1] Woolf AD, Akesson K (2001). Understanding the burden of musculoskeletal conditions. The burden is huge and not reflected in national health priorities. Br Med J.

[2] Health Canada (2003). Arthritis in Canada: An Ongoing Challenge.

[3] Bombardier C, Hawker G, Mosher D (2011). The Impact of Arthritis in Canada: Today and Over the Next 30 Years. Arthritis Alliance of Canada.

[4] Brosseau L, MacLeay L, Robinson V, Wells G, Tugwell P (2003). Intensity of exercise for the treatment of osteoarthritis. Cochrane Database Syst Rev.

[5] Ottawa Panel (2005). Ottawa panel evidence-based clinical practice guidelines for therapeutic exercises and manual therapy in the management of osteoarthritis. Phys Ther.

[6] Zhang W, Moskowitz R, Nuki G, Abramson S, Altman R, Arden N, Bierma-Zeinstra S, Brandt K, Croft P, Doherty M, Dougados M, Hochberg M, Hunter D, Kwoh K, Lohmander L, Tugwell P (2007). OARSI recommendations for the management of hip and knee osteoarthritis, part I: critical appraisal of existing treatment guidelines and systematic review of current research evidence. Osteoarthritis Cartilage.

[7] Zhang W, Moskowitz R, Nuki G, Abramson S, Altman R, Arden N, Bierma-Zeinstra S, Brandt K, Croft P, Doherty M, Dougados M, Hochberg M, Hunter D, Kwoh K, Lohmander L, Tugwell P (2008). OARSI recommendations for the management of hip and knee osteoarthritis, part II: OARSI evidence-based, expert consensus guidelines. Osteoarthritis Cartilage.

[8] Tremblay MS, Warburton DE, Janssen I, Paterson DH, Latimer AE, Rhodes RE, Kho ME, Hicks A, Leblanc AG, Zehr L, Murumets K, Duggan M (2011). New Canadian physical activity guidelines. Appl Physiol Nutr Metab.

[9] Dunlop DD, Song J, Semanik PA, Chang RW, Sharma L, Bathon JM, Eaton CB, Hochberg MC, Jackson RD, Kwoh CK, Mysiw WJ, Nevitt MC, Hootman JM (2011). Objective physical activity measurement in the osteoarthritis initiative: are guidelines being met?. Arthritis Rheum.

[10] Wallis J, Webster K, Levinger P, Taylor N (2013). What proportion of people with hip and knee osteoarthritis meet physical activity guidelines? A systematic review and meta-analysis. Osteoarthritis Cartilage.

[11] Gyurcsik NC, Brawley LR, Spink KS, Brittain DR, Fuller DL, Chad K (2009). Physical activity in women with arthritis: examining perceived barriers and self-regulatory efficacy to cope. Arthritis Rheum.

[12] der Ananian C, Wilcox S, Saunders R, Watkins K, Evans A (2006). Factors that influence exercise among adults with arthritis in three activity levels. Prev Chronic Dis.

[13] Henchoz Y, Zufferey P, So A (2013). Stages of change, barriers, benefits, and preferences for exercise in RA patients: a cross-sectional study. Scand J Rheumatol.

[14] Michie S, Richardson M, Johnston M, Abraham C, Francis J, Hardeman W, Eccles MP, Cane J, Wood CE (2013). The behavior change technique taxonomy (v1) of 93 hierarchically clustered techniques: building an international consensus for the reporting of behavior change interventions. Ann Behav Med.

[15] Lyons EJ, Lewis ZH, Mayrsohn BG, Rowland JL (2014). Behavior change techniques implemented in electronic lifestyle activity monitors: a systematic content analysis. J Med Internet Res.

[16] Li LC, Sayre EC, Xie H, Clayton C, Feehan LM (2017). A community-based physical activity counselling program for people with knee osteoarthritis: feasibility and preliminary efficacy of the track-OA study. JMIR Mhealth Uhealth.

[17] Lyons EJ, Swartz MC, Lewis ZH, Martinez E, Jennings K (2017). Feasibility and acceptability of a wearable technology physical activity intervention with telephone counseling for mid-aged and older adults: a randomized controlled pilot trial. JMIR Mhealth Uhealth.

[18] Li LC, Sayre EC, Xie H, Falck RS, Best JR, Liu-Ambrose T, Grewal N, Hoens AM, Noonan G, Feehan LM (2018). Efficacy of a community-based technology-enabled physical activity counseling program for people with knee osteoarthritis: proof-of-concept study. J Med Internet Res.

[19] Altman R, Asch E, Bloch D, Bole G, Borenstein D, Brandt K, Christy W, Cooke TD, Greenwald R, Hochberg M (1986). Development of criteria for the classification and reporting of osteoarthritis. Classification of osteoarthritis of the knee. Diagnostic and therapeutic criteria committee of the american rheumatism association. Arthritis Rheum.

[20] Thomas S, Reading J, Shephard RJ (1992). Revision of the physical activity readiness questionnaire (PAR-Q). Can J Sport Sci.

[21] Gupta A, Tong X, Shaw CD, Li LC, Feehan LM (2017). FitViz: A Personal Informatics Tool for Self-Management of Rheumatoid Arthritis. Proceedings of the International Conference on Human-Computer Interaction.

[22] Rollnick S, Miller WR, Butler CC (2008). Motivational Interviewing in Health Care: Helping Patients Change Behavior.

[23] Gutnick D, Reims K, Davis C, Gainforth H, Jay M, Cole S (2014). Brief action planning to facilitate behavior change and support patient self-management. J Clin Outcomes Manag.

[24] Tierney M, Fraser A, Purtill H, Kennedy N (2013). Study to determine the criterion validity of the sensewear armband as a measure of physical activity in people with rheumatoid arthritis. Arthritis Care Res (Hoboken).

[25] Holsgaard-Larsen A, Roos EM (2012). Objectively measured physical activity in patients with end stage knee or hip osteoarthritis. Eur J Phys Rehabil Med.

[26] Feehan LM, Goldsmith CH, Leung AY, Li LC (2016). Sensewearmini and actigraph GT3X accelerometer classification of observed sedentary and light-intensity physical activities in a laboratory setting. Physiother Can.

[27] Almeida GJ, Wasko MC, Jeong K, Moore CG, Piva SR (2011). Physical activity measured by the SenseWear Armband in women with rheumatoid arthritis. Phys Ther.

[28] Troiano RP, Berrigan D, Dodd KW, Mâsse LC, Tilert T, McDowell M (2008). Physical activity in the United States measured by accelerometer. Med Sci Sports Exerc.

[29] Ainsworth BE, Haskell WL, Herrmann SD, Meckes N, Bassett Jr DR, Tudor-Locke C, Greer JL, Vezina J, Whitt-Glover MC, Leon AS (2011). Compendium of Physical Activities Tracking Guide. Google Sites.

[30] Owen N (2012). Sedentary behavior: understanding and influencing adults' prolonged sitting time. Prev Med.

[31] Dunstan DW, Kingwell BA, Larsen R, Healy GN, Cerin E, Hamilton MT, Shaw JE, Bertovic DA, Zimmet PZ, Salmon J, Owen N (2012). Breaking up prolonged sitting reduces postprandial glucose and insulin responses. Diabetes Care.

[32] Latouche C, Jowett JB, Carey AL, Bertovic DA, Owen N, Dunstan DW, Kingwell BA (2013). Effects of breaking up prolonged sitting on skeletal muscle gene expression. J Appl Physiol (1985).

[33] Howard BJ, Fraser SF, Sethi P, Cerin E, Hamilton MT, Owen N, Dunstan DW, Kingwell BA (2013). Impact on hemostatic parameters of interrupting sitting with intermittent activity. Med Sci Sports Exerc.

[34] Roos EM, Roos HP, Lohmander LS, Ekdahl C, Beynnon BD (1998). Knee injury and osteoarthritis outcome score (KOOS)--development of a self-administered outcome measure. J Orthop Sports Phys Ther.

[35] Roos EM, Roos HP, Ekdahl C, Lohmander LS (1998). Knee injury and osteoarthritis outcome score (KOOS)--validation of a Swedish version. Scand J Med Sci Sports.

[36] Petkov J, Harvey P, Battersby M (2010). The internal consistency and construct validity of the partners in health scale: validation of a patient rated chronic condition self-management measure. Qual Life Res.

[37] Rhodes RE, Courneya KS, Blanchard CM, Plotnikoff RC (2007). Prediction of leisure-time walking: an integration of social cognitive, perceived environmental, and personality factors. Int J Behav Nutr Phys Act.

[38] Rhodes RE, Blanchard CM, Courneya KS, Plotnikoff RC (2009). Identifying belief-based targets for the promotion of leisure-time walking. Health Educ Behav.

[39] Manea L, Gilbody S, McMillan D (2012). Optimal cut-off score for diagnosing depression with the patient health questionnaire (PHQ-9): a meta-analysis. Can Med Assoc J.

[40] Gardner B, de Bruijn G, Lally P (2011). A systematic review and meta-analysis of applications of the self-report habit index to nutrition and physical activity behaviours. Ann Behav Med.

[41] Gardner B, Abraham C, Lally P, de Bruijn G (2012). Towards parsimony in habit measurement: testing the convergent and predictive validity of an automaticity subscale of the self-report habit index. Int J Behav Nutr Phys Act.

[42] Ory M, Resnick B, Jordan PJ, Coday M, Riebe D, Garber CE, Pruitt L, Bazzarre T (2005). Screening, safety, and adverse events in physical activity interventions: collaborative experiences from the behavior change consortium. Ann Behav Med.

[43] (2019). Rate Limits. Fitbit SDK.

[44] Diggle PG, Heagerty P, Liang KY, Zeger S (2013). Analysis of Longitudinal Data (Oxford Statistical Science Series - 25).

[45] (2014). iHealth: How Consumers Are Using Tech to Stay Healthy. The Nielsen Company.

[46] Osthoff AR, Niedermann K, Braun J, Adams J, Brodin N, Dagfinrud H, Duruoz T, Esbensen BA, Günther KP, Hurkmans E, Juhl CB, Kennedy N, Kiltz U, Knittle K, Nurmohamed M, Pais S, Severijns G, Swinnen TW, Pitsillidou IA, Warburton L, Yankov Z, Vlieland TP (2018). 2018 EULAR recommendations for physical activity in people with inflammatory arthritis and osteoarthritis. Ann Rheum Dis.

[47] Kolasinski SL, Neogi T, Hochberg MC, Oatis C, Guyatt G, Block J, Callahan L, Copenhaver C, Dodge C, Felson D, Gellar K, Harvey WF, Hawker G, Herzig E, Kwoh CK, Nelson AE, Samuels J, Scanzello C, White D, Wise B, Altman RD, DiRenzo D, Fontanarosa J, Giradi G, Ishimori M, Misra D, Shah AA, Shmagel AK, Thoma LM, Turgunbaev M, Turner AS, Reston J (2020). 2019 American college of rheumatology/arthritis foundation guideline for the management of osteoarthritis of the hand, hip, and knee. Arthritis Rheumatol.

[48] Ellingson LD, Lansing JE, DeShaw KJ, Peyer KL, Bai Y, Perez M, Phillips LA, Welk GJ (2019). Evaluating motivational interviewing and habit formation to enhance the effect of activity trackers on healthy adults' activity levels: randomized intervention. JMIR Mhealth Uhealth.

[49] O’Brien MW, Shields CA, Campbell KL, Crowell SJ, Fowles JR (2019). Perceptions and practices of providing physical activity counselling and exercise prescriptions among physiotherapists in Nova Scotia. Phys Can.

[50] Borrelli B, Sepinwall D, Ernst D, Bellg AJ, Czajkowski S, Breger R, DeFrancesco C, Levesque C, Sharp DL, Ogedegbe G, Resnick B, Orwig D (2005). A new tool to assess treatment fidelity and evaluation of treatment fidelity across 10 years of health behavior research. J Consult Clin Psychol.

[51] Lobelo F, Stoutenberg M, Hutber A (2014). The exercise is medicine global health initiative: a 2014 update. Br J Sports Med.

